# Effects of Low-Intensity Aerobic Exercise on Neurophysiological and Behavioral Correlates of Cognitive Function

**DOI:** 10.3390/bs13050401

**Published:** 2023-05-10

**Authors:** Ryan L. Olson, David J. Cleveland, Melissa Materia

**Affiliations:** Department of Kinesiology, Health Promotion and Recreation, University of North Texas, Denton, TX 76203, USA

**Keywords:** aerobic exercise, P3, cognitive function, ERP, EEG

## Abstract

Acute aerobic exercise exerts a small beneficial effect on cognition. Previous research primarily examines cognitive changes following a bout of exercise, while little is currently known about changes in cognitive performance during exercise. The primary purpose of this study was to examine the effects of low-intensity cycling on cognitive function indexed by behavioral (response accuracy; reaction time) and neurocognitive (P3 mean amplitude; P3 centroid latency) responses. Twenty-seven (*M*_age_ = 22.9 ± 3.0 years old) individuals were counterbalanced into low-intensity exercise (EX) and seated control (SC) conditions spread across two testing sessions. During each condition, participants completed a 10 min resting baseline period, 20 min of either sustained cycling or seated rest, and a 20 min recovery period. Primary outcomes were assessed at 10 min intervals (five blocks total) throughout each condition via a modified visual oddball task while electroencephalography (EEG) responses were measured. Across time blocks, both conditions exhibited faster reaction times on frequent trials but reduced accuracy to rare trials, suggesting a speed–accuracy tradeoff. There were no differences between conditions in P3 centroid latency, whereas a significant reduction in P3 amplitude was observed during the 20 min exercise period compared to the control condition. Taken together, results suggest that exercise at lower doses may have minimal influence on behavioral outcomes of cognitive performance but may impact more basic measures of brain function. Information gathered from this study may aid in the development of appropriate exercise prescriptions for populations looking to specifically target cognitive function deficits.

## 1. Introduction

Acute aerobic exercise exerts a small beneficial effect on cognition [1,2,3,4]. This conclusion was drawn following a number of empirical investigations, systematic reviews, and meta-analyses indicating the general and specific effects of exercise on cognitive function [5,6,7,8,9,10]. Most previous research examines cognitive changes following acute bouts of exercise, while little is currently known about changes in cognitive performance that occur during exercise. Exploring this relationship may be especially important for understanding the neurobiological mechanisms underlying the alterations we see following exercise. The limited research that has been conducted suggests either positive [3,8,11,12], negative [13,14], or no [15] effects on cognitive performance during exercise.

Studies examining the effects of exercise on cognition have traditionally utilized end-state measures of overt behavioral task performance such as response accuracy and reaction time. While this information has undoubtedly laid the groundwork in the area of exercise and cognition, these outcomes tell us very little about the subtle aspect of cognitive processing that may be influenced by exercise, as well as providing little information about the potential mechanisms underlying the effects of exercise on brain function. With the advent of advanced functional neuroimaging techniques, researchers are now able to measure brain function safely and accurately during exercise through electroencephalography (EEG) [15,16,17]. EEG is used to reveal specific temporal and general spatial properties of neural activity during cognitive tasks that cannot be examined with traditional behavioral measures [18]. We can further decompose the continuous EEG signal via the event related potential (ERP) technique, which may provide researchers with valuable information about the processes that occur before, during, and after the execution of a behavior. ERPs represent post-synaptic voltage fluctuations that are time-locked to a specific event, such as the onset of a stimulus or the execution of a manual response. Several components of the ERP signal have been identified and are thought to reflect the sensory, cognitive, affective, and motor processes elicited by a stimulus [19]. The P3 component has received a bulk of researcher attention in the exercise–cognition literature. The amplitude and latency of P3, named for its location within the ERP (i.e., third positive peak), is commonly measured in cognitive neuroscience as an index of attentional resource allocation during stimulus engagement and stimulus classification speed, respectively [20,21,22]. The P3 component is a stimulus-locked ERP observed approximately 300–800 ms following stimulus onset and has been instrumental in continuing our knowledge base of cognition and brain function both during and following exercise [8,12,14,23,24,25].

In the few studies that have incorporated ERPs during exercise, equivocal findings were reported. In one of the most recent studies, Scanlon and colleagues (2017) had participants (*N* = 14) complete an auditory oddball task while EEG was recording during pre-exercise, exercise, and post-exercise conditions. Participants sat on a stationary bike during pre- and post-exercise conditions while the exercise condition consisted of pedaling at low intensity. The authors selected the oddball paradigm, used to assess working memory, attention, and inhibitory control, because it elicits a robust and isolated P3 component. Results indicated no significant difference in P3 between the biking and sitting conditions. These findings are in line with previous studies showing no differences in P3 during exercise [26,27,28,29,30], though the authors did note there was a marginal decrease in P3 amplitude from pre- to post-exercise. They suggested that the effect may be explained by habituation effects whereby the P3 dissipates over time as a task becomes less novel. No specific information was provided on exercise intensity or duration, but the authors did indicate that participants were asked to pedal slowly and consistently, without exerting themselves or raising their heart rate. Additionally, participants completed 750 trials (three blocks of 250 trials separated by a self-paced rest period >0.5 s) before, during, and after pedaling. Each trial consisted of a random length pre-tone interval between 500–1000 ms followed by tone onset lasting 16 ms. Estimated time for each block of 250 trials ranged between 2.15–4.23 min (total estimated exercise duration equal to 6.45–12.69 min). Thus, the exercise would be classified as very light intensity taking place over a period of time (i.e., <15 min) not typically shown to improve cognitive function.

Contrary to these findings, Yagi et al. (1999) found reduced P3 amplitudes in individuals completing two versions of the oddball paradigm during exercise compared to rest and recovery periods. Participants performed an auditory and visual oddball paradigm during three conditions: rest, exercise on a recumbent bicycle ergometer, and a recovery period. Participants cycled at approximately 70% of maximal age predicted heart rate which is equivalent to a moderate intensity. For the first block of testing, half the participants (*n* = 12) completed the auditory oddball task during each condition. Next, the same participants completed the second block of testing with the visual oddball task during each condition. The second group of participants completed the oddball paradigms in reverse order during each condition. Similar to previous research, there was a main effect of exercise on reaction time such that faster response times were recorded during exercise while response accuracy was reduced during exercise. Relative to P3, latency and amplitude were decreased (i.e., faster and lower, respectively) during exercise compared to rest and recovery periods. The authors suggest that reductions in P3 amplitude may be due to participants treating exercise as a secondary task requiring a larger fraction of limited attentional resources (e.g., dual-task interference) such that resources typically reserved for cognitive processing were being re-directed to the body to maintain exercise. Furthermore, the reduction in P3 latency was explained as being due to exercise induced arousal whereby increased arousal via exercise reduces cognitive processing speed.

Most studies examining ERPs during exercise find increases in P3 amplitude and decreases in P3 latency [12,14,31,32]. For example, Olson and colleagues (2016) examined ERP responses to a flanker task during low-intensity (40% VO_2_ peak) exercise, moderate-intensity (60% VO_2_ peak) exercise, and control conditions. Researchers found increased P3 amplitude across centro-parietal electrode sites during both exercise intensities relative to the control condition. There we no differences between conditions. These results suggest exercising while completing a cognitive task may increase attentional resources required to successfully perform [33]. Specifically, the reported increases in P3 amplitude across both trial types (i.e., congruent and incongruent stimuli) may have been due to greater upregulation of cognitive control and attentional resources necessary for successful task completion. However, this particular study employed a modified flanker paradigm with the presentation of equiprobable stimuli. Equiprobable stimuli presentation would likely reduce P3 responses due to the heavy influence of probability on amplitude [34]. Furthermore, the flanker paradigm is traditionally used for assessing inhibitory cognitive control and response monitoring via the N2 and ERN ERP components, respectively, and may not be an appropriate task to utilize when assessing P3. Pontifex and Hillman (2007) also assessed ERP responses during rest and exercise conditions, instructing participants to cycle at steady state (60% of maximal heart rate) for approximately 6.5 min during the exercise condition. They found increases in P3 amplitude and reductions in latency across frontal and lateral electrode sites during an equiprobable flanker paradigm. The authors interpreted the results through a cortical inefficiency theory, suggesting that during stimulus engagement, exercise leads to delays in stimulus evaluation and classification speed, which may reduce interference control. Furthermore, the authors indicated that the increases in P3 amplitude were likely due to increased attentional resources required to complete the cognitive task or that the resources were inefficient rather than there being an insufficient amount. Finally, Grego et al. (2004) had trained cyclists exercise at moderate intensity (~66% VO_2_ max) for 180 min to study the effects of fatigue on P3 during an auditory oddball paradigm. There were no P3 amplitude differences between rest and exercise conditions during the first (3 min) and second (36 min) time points. As time progressed, P3 amplitude increased during the third (72 min) and fourth (108 min) time points. P3 amplitude was later diminished during the fifth (144 min) and sixth (180 min) time points. The authors suggested that the increases in P3 amplitude between the third and fourth time points were reduced at the later time points through the combined effects of arousal and central fatigue mechanisms during prolonged exercise. Despite the mixed results and variety of proposed mechanisms, there is general consensus that cognitive function, measured by behavioral and neuroelectric performance, is modifiable during exercise.

The purpose of this study was to examine the effects of low-intensity cycling on cognitive function measures of behavioral (response accuracy; reaction time) and neurocognitive (P3 amplitude; P3 centroid latency) performance in college-aged students. It was hypothesized that low-intensity exercise would reduce reaction time (i.e., become faster) and have no effect on response accuracy relative to a seated control condition. Furthermore, it was predicted that the exercise condition would display a significant increase in P3 amplitude and reduction in P3 centroid latency compared to the control condition.

## 2. Materials and Methods

### 2.1. Participants

Twenty-seven (10 females; 17 males) college-aged individuals (*M*_age_ = 22.9 ± 3.0) were recruited from the local university via recruitment emails and flyers. Inclusion criteria included: men and women, aged 18–35 years, and no physical limitations or contraindications to exercise. Participants were excluded from participation if they met one or more of the following criteria: current or present history of cardiovascular disease, past or present history of psychiatric or neurological disorder, currently taking medications that would prevent them from completing moderate-to-vigorous intensity exercise, and/or pregnancy or considering becoming pregnant in women. Due to the within-subjects design, enrolled participants were screened for regular sleep patterns, stimulant use (e.g., caffeine and tobacco), meal consumption, exercise participation, stress levels, and current mood prior to each testing session. Any subject who provided irregular responses at the beginning of either session relative to their normal responses were re-scheduled. The Institutional Review Board at the University of North Texas approved research procedures and all participants provided written informed consent prior to data collection.

### 2.2. Measures

#### 2.2.1. General Medical History

A complete health and medical history were obtained during the familiarization day using a self-reported medical history questionnaire. The form assessed family history or presence of disease, medical symptoms, past surgeries, tobacco/alcohol use, and prior and current medication use. Participant height and weight were also recorded on the form and used for the calculation of body mass index (BMI). Participants were also asked to complete the Physical Activity Readiness Questionnaire (PAR-Q) [35], a screening tool used to ensure safety for participating in the exercise condition. Anyone responding “No” to any question within the PAR-Q was required to obtain medical clearance from their doctor prior to participating. Lastly, due to the potential influence of mental health status on ERP responses, participants completed the Beck Depression Inventory (BDI) [36], State-Trait Anxiety Inventory (STAI) [37], and Perceived Stress Scale (PSS) [38].

#### 2.2.2. Heart Rate (HR) and Intensity

Heart rate (HR) was monitored continuously throughout the test sessions with a Polar S810 HR monitor and transmitter (Polar Electro, Kemele, Finland). HR data points were collected at minute 0 and every 10 min thereafter to ensure participants maintained a relative exercise intensity that fell within the prescribed zone. In order to standardize workload intensity between conditions, low-intensity exercise was defined as maintaining a HR range between 57–63% of age-predicted maximum heart rate (HR_max_). HR_max_ was calculated based on the American College of Sports Medicine (ACSM) guidelines (i.e., 220 − age = HR_max_) for establishing exercise intensity zones [39].

#### 2.2.3. Ratings of Perceived Exertion (RPE)

The in-task ratings of perceived exertion (RPE) were measured using Borg’s 15-point scale [40], which ranges from 6 to 20 with verbal anchors at 7 (very, very light), 9 (very light), 11 (fairly light), 13 (somewhat hard), 15 (hard), 17 (very hard), and 19 (very, very hard). Meta-analytic findings indicate strong validity with common physiological measures of exertion and intensity [41]. The validity of the RPE scale in terms of its correlation with standard physiological indices (e.g., blood lactate, oxygen uptake, respiratory exchange ratio) has been previously demonstrated (*r* = 0.80 to 0.95) [42]. The scale also displays both high intratest (*r* = 0.93) and retest (*r* = 0.83 to 0.94) reliability [42].

#### 2.2.4. Oddball Paradigm

Participants completed a modified version of a visual oddball paradigm [43] to measure sustained attention and working memory capacity. The stimuli consisted of 3 cm tall × 3 cm long black letters (B, C, D, E, F) and digits (2, 3, 4, 5, 6) presented focally on a computer screen following a continuous fixation point. The monitor was viewed at a distance of approximately 100 cm with vertical and horizontal visual angles of 1.7 × 1.7 degrees, respectively. During experimental sessions (days 2 and 3), participants completed a brief training block (approximately 25 trials) where response feedback was provided. This was used to remind participants of the cognitive task they completed during familiarization (day 1). Next, participants completed two blocks of 60 trials without feedback at five separate time points: 5, 15, 25, 35, and 45 min (600 total trials). Each stimulus was presented in black font color on a light grey background for 100 ms. To avoid potential anticipatory responses, a random intertrial interval (ITI) ranging between 800–1200 ms was implemented prior to each stimulus presentation. Participants were instructed to respond with their right hand for digits and left hand for letters, then instructed to switch (right for letters and left for digits) on the next block of trials to prevent response mapping. The order of response instructions was counterbalanced between the five time points. The oddball task consisted of two blocks, one block of trials where digits appeared 80% of the time and letters appeared 20% of the time and a second block of trials where digits appeared 20% of the time and letters appeared 80% of the time. Blocks were counterbalanced throughout the session to prevent practice effects. In total, each assessment consisted of 120 trials with a 20 s rest period between each 60-trial block.

#### 2.2.5. P3 Event-Related Potential (ERP)

Continuous EEG was recorded from 28 monopolar electrode sites (FP1, FP2, F3, F4, F7, F8, FC1, FC2, FC5, FC6, C3, C4, CP1, CP2, CP5, CP6, TP9, TP10, P3, P4, P7, P8, O1, O2, Fz, Cz, Pz, Oz) arranged in accordance with the international 10–20 system [44] using a Brain Vision actiCap with active electrodes and actiCHamp amplifier system (Brain Products GmbH; Munich, Germany). A bipolar arrangement of vertical (above and below the left eye) and horizontal (approximately 1 cm lateral to the outer canthus of each eye) electrodes were used to measure electrooculogram (EOG) activity for eye movements and artifact. Continuous data was initially referenced to the vertex electrode (Cz) and digitized at 500 Hz with a 24-bit analog-to-digital converter. Impedances were assessed prior to each testing block and maintained below 10 kΩ throughout the session. Data were exported from PyCorder (version 1.0.9) to the ERP Principal Component Analysis (PCA) toolkit [45] and bandpass filtered using a 2nd order infinite impulse response (IIR) Butterworth filter with a low-pass frequency of 30 Hz and high-pass frequency of 0.1 Hz. Data was then manually inspected for large movement-related artifacts (e.g., blink artifact, eye movements, and muscle activity). Prior to segmenting, independent component analysis (ICA) was applied to continuous data for the detection and removal of eye blinks. Stimulus-locked epochs were then created from 100 ms pre- to 1000 ms post-stimulus onset. Artifact correction was then applied to segmented trials to remove residual eye blinks and saccades. ICA blink templates were generated within the PCA Toolkit, with one generated from the dataset of all subjects and one default template provided by the toolkit author. ICA components that correlated at 0.9 with scalp topographies of either blink template were removed. Additionally, trials with a difference of 100 μV between minimum and maximum values in that trial or channels differing in the segment by more than 30 μV from the neighboring six closest channels were marked as bad and removed. Trials with >10% of channels marked as bad were also removed. The remaining bad channels were corrected through spherical interpolation obtained from “good” channels of the scalp voltage field within each segment. Lastly, epochs were re-referenced to the left and right mastoids [46,47], averaged by trial type, and baseline corrected using the 100 ms pre-stimulus period. Only correct trials were averaged to analyze P3 component amplitude and latency. Due to the scalp distribution of P3, and consistent with previous ERP research [21], amplitude and latency were assessed at centro-parietal (CP1, CP2, Cz, Pz) electrode sites. Amplitude was measured as the mean amplitude between rare and frequent stimuli within an a priori time window of 300–700 ms post-stimulus onset [48] for the grand averaged waveform while latency was measured as the maximal centroid latency between rare and frequent stimuli during the same time window.

### 2.3. Procedures

**Figure 1:**
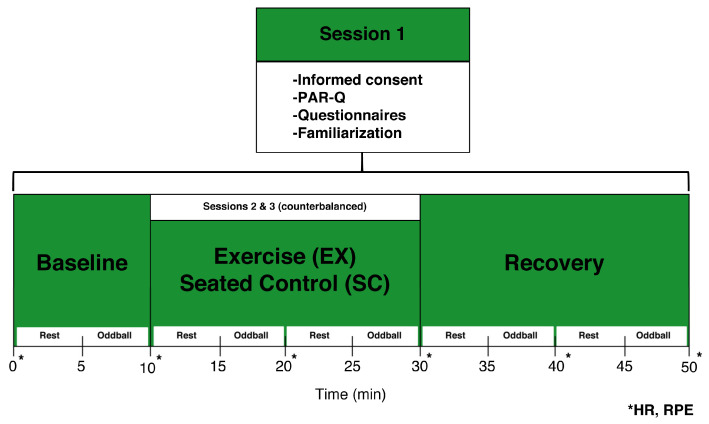
Study Diagram.

Participants visited the laboratory on three separate occasions at approximately the same time of day separated by at least 24 h between sessions (see Figure 1). On day 1 (familiarization), participants provided written informed consent and were asked to complete the PAR-Q and a brief health history form. Next, participants were familiarized with the exercise equipment, EEG recording chamber, and cognitive testing. Briefly, the participant sat on the recumbent cycle ergometer approximately 100 cm from the computer monitor. Participants were asked to pedal for 5 min at a self-selected pace and resistance to become familiar with the mechanics of the equipment. Adjustments in equipment distance were made throughout the 5 min period and were recorded for use during the remaining test days. Additionally, participants completed a practice oddball task consisting of 50 trials to ensure they understood the directions. Feedback indicating response accuracy and reaction time was provided on practice trials in order for participants to answer as quickly and accurately as possible.

On days 2 and 3 (experimental sessions), participants were counterbalanced into a low-intensity exercise (EX) or seated control (SC) condition. The recumbent bike was adjusted to the previously recorded position for both sessions. Participants were then fitted with a polar S810 HR monitor and EEG cap. Next, participants were seated on the recumbent bike and asked to place their feet in the pedal straps. During the EX condition, participants pedaled at a self-selected pace for 20 min while resistance was adjusted to match a low-intensity range based on HR and RPE values previously calculated during the familiarization session. During the SC condition, participants left their feet on the pedals and sat quietly during the same 20 min period. Overall, participants completed 10 min rest, 20 min test, and 20 min recovery periods with a 5 min block of neurocognitive testing taking place every 10 min (see Figure 1). Measures of RPE, and HR were recorded at minute 0 and every 10 min thereafter until the end of the test session. Upon completion of both test sessions, participants were debriefed on the purpose of the study.

### 2.4. Data Analysis

Descriptive statistics were first performed on participant demographics data using SPSS Statistical Software version 24 (SPSS Inc., Chicago, IL, USA). A within-subjects experimental design was utilized to examine the effects of low-intensity exercise on primary outcomes of neurocognitive function. All outcome measures were assessed throughout each condition at either five (oddball; P3) or six (HR; RPE) time points. Repeated measures analysis of variance (RM-ANOVA) was used for P3 amplitude and latency, response accuracy, reaction time, HR, and RPE with a 2-tailed alpha level of 0.05 for all statistical tests. As a manipulation check of exercise intensity, a 2 (Condition: EX, SC) × 6 (Time Block: 0, 1, 2, 3, 4, 5) RM-ANOVA was conducted to compare HR and RPE across conditions. This analysis expectedly produced a quadratic trend in HR and RPE from rest to exercise and exercise to recovery only in the EX condition, with no change observed in the SC condition. Behavioral performance measures of reaction time and response accuracy were submitted to a 2 (Condition: EX, SC) × 5 (Time Block: 1, 2, 3, 4, 5) × 2 (Trial Type: Rare, Frequent) RM-ANOVA. Trials with reaction time and accuracy scores beyond the individual mean ± 3 SD for each trial type were excluded to reduce the potential effect of outliers. Based on previous research [12,14] and due to P3 being most robust at centro-parietal regions [34,49], statistical analyses for P3 amplitude and centroid latency were performed using an a priori 4-electrode region of interest (ROI) averaged across centro-parietal electrode sites (Cz, CP1, CP2, Pz). Accordingly, mean P3 amplitude and centroid latency data were submitted to a 2 (Condition: EX, SC) × 5 (Time Block: 1, 2, 3, 4, 5) × 2 (Trial Type: Rare, Frequent) RM-ANOVA. All planned comparisons and post hoc analyses were conducted using Bonferroni corrected *t* tests. Effect sizes (ESs) are presented as partial eta squared (η^2^_p_) for ANOVA results.

## 3. Results

Preliminary analyses revealed no significant sex differences in BMI or age (see Table 1). Similarly, there were no significant sex differences in ratings of perceived exertion or heart rate responses throughout the test session. Subsequent analyses were collapsed across sex. The total number of correct trials included in the grand averaged ERP waveforms did not differ between exercise (541 trials) and control (535 trials) conditions. Similarly, the total number of trials did not differ across time blocks 1 (219), 2 (218), 3 (211), 4 (215), and 5 (212). Initially, 50 participants were recruited to participate in the study. A total of 23 participants were removed from the final analysis. A total of 18 participants were removed due to incomplete data whereby participants would complete either session 1 or sessions 1 and 2, but not session 3. An additional five participants were removed due to irregular EEG recordings contaminated with excessive eye blinks and movement artifact. Therefore, a total of 27 participants remained in the following analyses.

### 3.1. Measures

#### 3.1.1. Heart Rate (HR)

**Figure 2:**
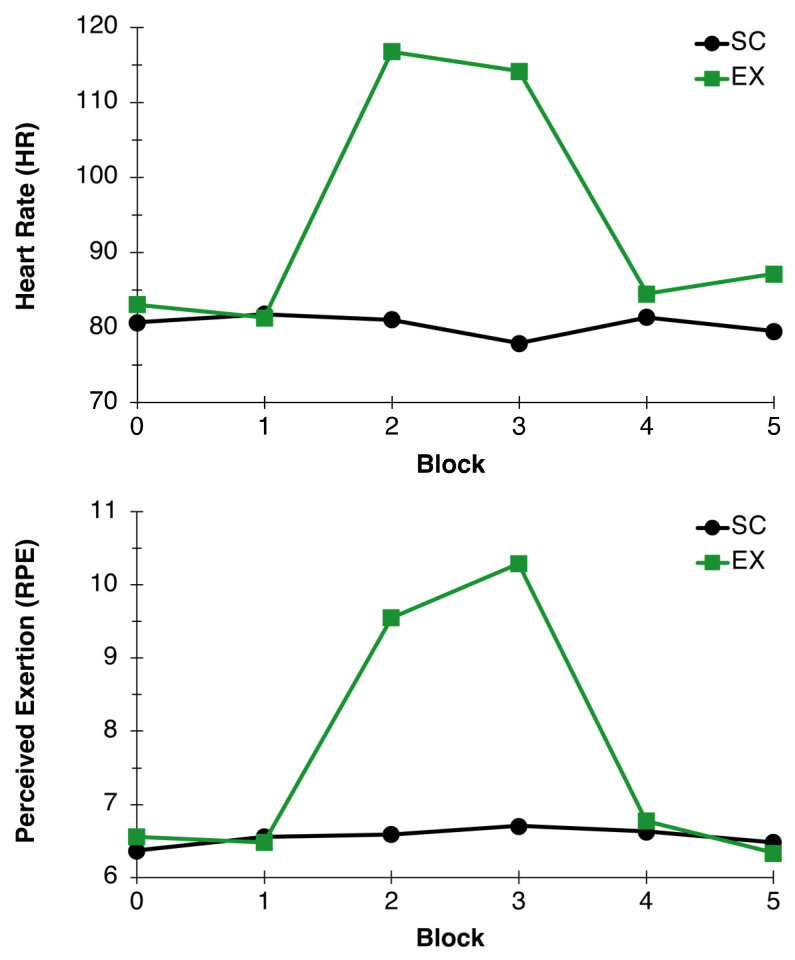
Heart Rate and Perceived Exertion. Average heart rate (BPM; **top**) and perceived exertion (RPE; **bottom**) measured during blocks 0, 1, 2, 3, 4, and 5 for EX (green line) and SC (black line) conditions.

As expected, average HR during exercise fell within the appropriate 57–63% HR_max_ range (115.44 ± 11.52) for the exercise condition. Additionally, the two-factor RM-ANOVA for HR revealed main effects for Condition, *F*(1,26) = 64.55, *p* < 0.001, η^2^_p_ = 0.71, and Time, *F*(5,22) = 22.80, *p* < 0.001, η^2^_p_ = 0.84. These effects were superseded by a Condition x Time interaction, *F*(5,22) = 31.42, *p* < 0.001, η^2^_p_ = 0.88, such that HR was similar during the rest and recovery periods and higher during the exercise bout in the exercise condition compared to the control condition, confirming the prescribed intensity was met by participants in the exercise group (see Figure 2).

#### 3.1.2. Ratings of Perceived Exertion (RPE)

As Significant Condition, *F*(1,26) = 16.54, *p* < 0.001, η^2^_p_ = 0.39, and Time, *F*(5,22) = 13.52, *p* < 0.001, η^2^_p_ = 0.75, main effects were found for RPE. A Condition x Time interaction superseded these main effects, *F*(5,22) = 16.09, *p* < 0.001, η^2^_p_ = 0.79, indicating RPE was similar during the rest and recovery periods and higher during the exercise bout in the exercise condition compared to the control condition, further confirming the prescribed intensity was met by participants in the exercise group (see Figure 2).

#### 3.1.3. Response Accuracy and Reaction Time

**Figure 3:**
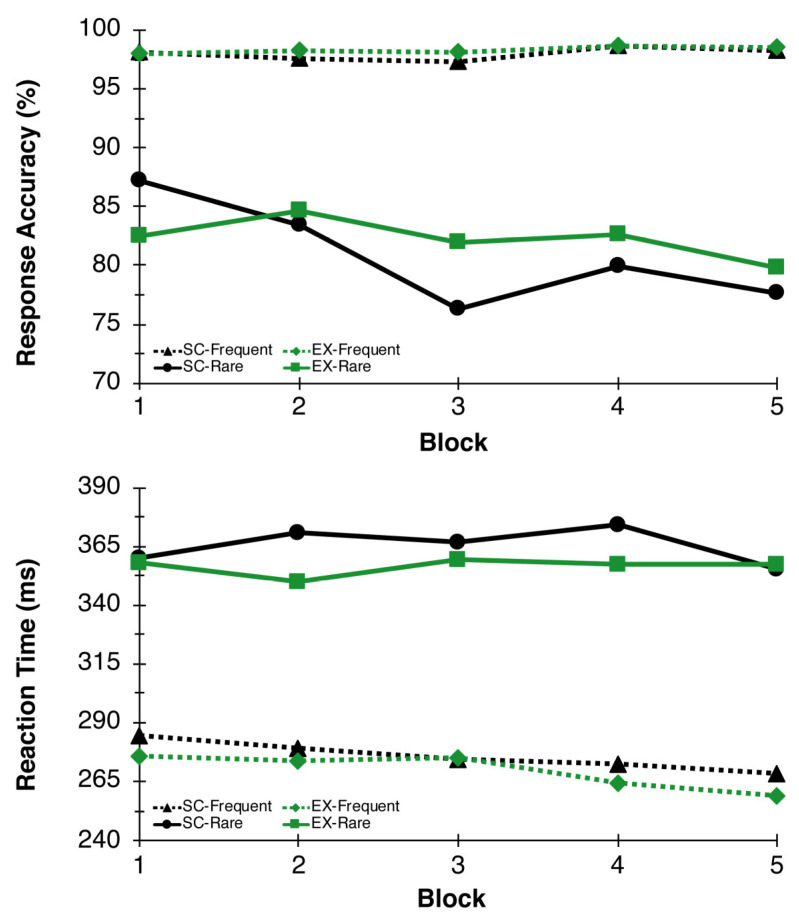
Response Accuracy and Reaction Time. Response accuracy (**top**) and reaction time (**bottom**) performance on the oddball paradigm during blocks 1, 2, 3, 4, and 5 for EX (green lines) and SC (black lines) conditions.

As expected, accuracy results revealed a significant Congruency main effect between frequent and rare trials, *F*(1,26) = 57.15, *p* < 0.001, η^2^_p_ = 0.69, indicating less accurate responses on rare (81.6 ± 2.3%) relative to frequent (98.2 ± 0.2%) trials. There was also a Time main effect nearing significance, *F*(4,23) = 2.72, *p* = 0.055, η^2^_p_ = 0.32, suggesting reductions in accuracy over time. The Congruency main effect was superseded by a Time x Congruency interaction, *F*(4,23) = 4.54, *p* = 0.008, η^2^_p_ = 0.44, such that rare trial accuracy reduced over time, while frequent trial accuracy remained steady throughout the test session. No additional main effects or interactions were found for response accuracy measures. For reaction time, a Congruency main effect was found, *F*(1,26) = 248.63, *p* < 0.000, η^2^_p_ = 0.91, such that reaction time to frequent trials was faster (272.8 ± 8.8 ms) compared to rare trials (361.0 ± 9.5 ms). No additional main effects or interactions were found for reaction time (see Figure 3).

#### 3.1.4. P3 Amplitude and Latency

**Figure 4:**
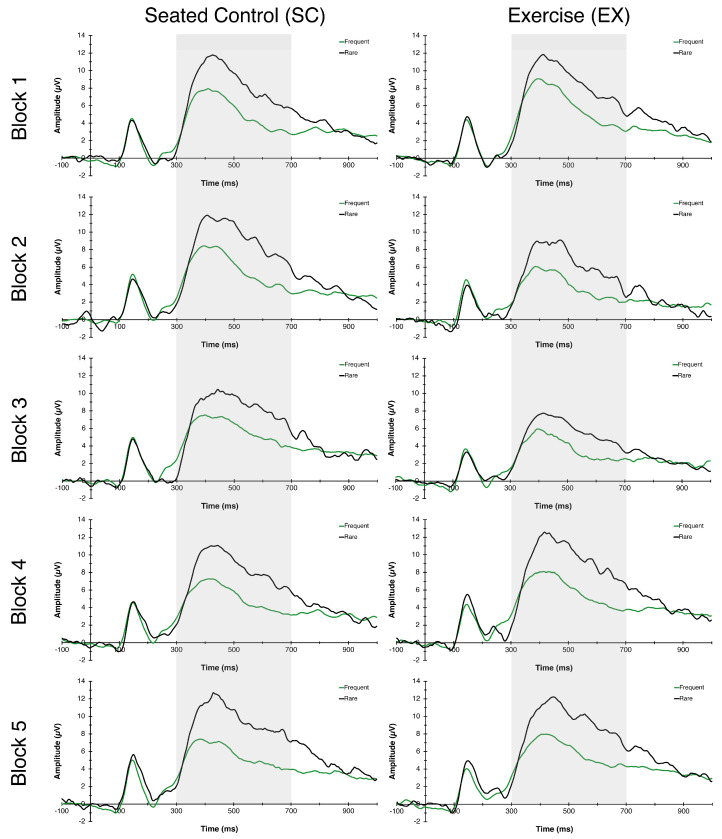
P3 Grand Average ERPs. Grand average P3 waveforms averaged across centro-parietal electrode sites (Cz, CP1, CP2, Pz) measured during blocks 1, 2, 3, 4, and 5 for SC (**left** column) and EX (**right** column) conditions. Frequent trials are represented by a green line, rare trials are represented by a black line, and the 300–700 ms P3 time window is represented in grey shading.

**Figure 5:**
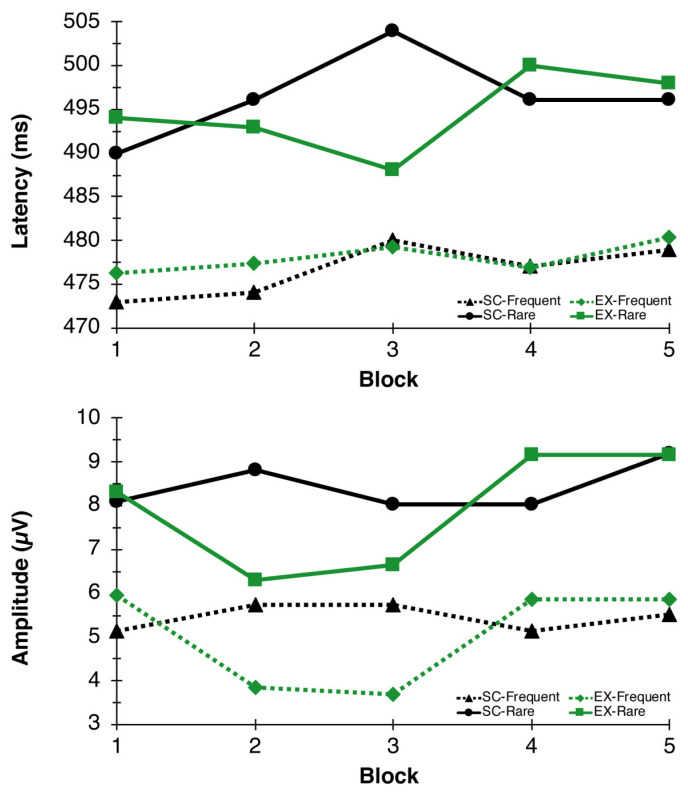
P3 Latency and Amplitude. P3 latency (**top**) and amplitude (**bottom**) responses to the oddball paradigm during blocks 1, 2, 3, 4, and 5 for EX (green lines) and SC (black lines) conditions.

The RM-ANOVA for P3 latency revealed a significant Congruency main effect, *F*(1,26) = 37.05, *p* = 0.006, η^2^_p_ = 0.59, indicating faster latency to frequent (477.45 ± 4.98 ms) compared to rare (496.04 ± 4.85 ms) trials. No additional main effects or interactions were found for P3 latency. For P3 amplitude, main effects of Time *F*(4,23) = 4.73, *p* = 0.006, η^2^_p_ = 0.45, and Congruency *F*(1,26) = 57.47, *p* < 0.000, η^2^_p_ = 0.69, were found. These main effects were superseded by a Condition × Time interaction, *F*(4,23) = 3.50, *p* = 0.023, η^2^_p_ = 0.38, indicating that P3 amplitudes, in general, were reduced during exercise (blocks 2 and 3) whereas they remained stable throughout the seated control condition (see Figure 4 and Figure 5).

## 4. Discussion

The primary aim of this study was to examine the effects of low-intensity cycling on cognitive function in college-aged students as indexed by behavioral performance (response accuracy, reaction time) and neurocognitive responses (P3 amplitude and latency) to the oddball paradigm. It was hypothesized that low-intensity exercise would significantly reduce reaction time and have minimal effect on response accuracy. While there were trends for reduced reaction time on rare trials, no significant differences emerged between the exercise and seated control conditions. Aside from this trend, there were no additional between-group effects observed for reaction time. In partial support of our hypotheses, results for response accuracy indicated no significant group differences, but there was an interaction whereby accuracy on the rare trials was reduced over time across both conditions. While several studies have found impairments in similar behavioral performance measures during exercise [12,13,14], not all studies are in agreement [24,50,51]. Differences between the current study and previous investigations may be due to methodological decisions such as exercise intensity and duration, cognitive task selection and difficulty, the use of multiple time blocks, and the population being studied.

Relative to neurocognitive performance, it was hypothesized that exercise would increase P3 amplitude and reduce P3 latency compared to the seated control condition. We found contrasting results compared to a number of previous investigations [12,14,21]. In particular, P3 amplitude responses resembled a quadratic trend where it was similar between conditions at baseline, suppressed during both blocks of exercise, and returned to baseline levels during the recovery period. Latency responses, on the other hand, were similar between conditions, with no significant changes over time. These findings are supported by previous research indicating decreases in P3 amplitude during moderate-intensity exercise [30] and self-selected, low-intensity walking [22]. Authors suggested that during exercise, the cognitive paradigm is treated like a secondary task requiring a larger fraction of limited attentional resources (i.e., distraction/dual-task interference). That is, participants are not only required to complete the task successfully, but they must also split attention to the exercise bout they are asked to perform. However, it is worth noting that Yagi and colleagues (1999) had their participants complete two versions of the oddball paradigm, auditory and visual, and that the conditions, exercise and control, were administered back-to-back without counterbalancing. Thus, the findings may be influenced by potential task, order, or residual exercise effects.

Findings from the current investigation are further supported by the transient hypofrontality theory [52,53,54], which posits that successful task performance during exercise results in a situation where attention is drawn away from the cognitive task to maintain the necessary metabolic, neuromuscular, and cardiovascular responses to sustain exercise. Similarly, it is proposed that there are limited attentional and information processing resources available in the brain [55,56], and these resources are especially susceptible to stressors such as exercise [57,58]. Thus, performing a cognitive task while exercising may increase the demand placed upon available neural resources of the prefrontal cortex. This would likely be due to the control of bodily movements required to sustain exercise in addition to the cognitive demands required by the task [13]. The resources typically reserved for successful task completion would be shifted toward maintaining exercise demands, and this reduction in neural resources may eventually lead to reduced cognitive performance. To further understand this process, it is important for researchers to focus on accurately measuring the neural operations that mediate these complex cognitive processes.

Contrasting the current findings, a similarly designed study found increases in P3 amplitude during sustained low- and moderate-intensity exercise compared to a seated control condition [12]. However, key differences in exercise dose and cognitive task selection may explain these divergent results. For example, the use of a flanker task, which is traditionally used for assessing N2 and ERN ERP components, may require greater attentional resources to successfully complete due to the complexity of the task. Reaction time and response accuracy results from the study also suggested a potential speed accuracy trade-off on the most difficult incongruent trials of the flanker task. The lack of reaction time and accuracy findings in the current investigation may be partially due to the use of a simple oddball paradigm that presented fewer rare trials (20%) relative to the more complex incongruent flanker stimuli (50%). A similar study by Vogt et al. (2015) found an increase in P3 amplitudes to a mental arithmetic test that was completed during a moderate-intensity bout of self-paced cycling in a virtual environment. The authors found that P3 responses were only increased during exercise within the virtual environment, with no changes being observed during exercise alone. Moreover, no significant differences were observed in behavioral performance measures between exercise and control conditions. As with previous interpretations, it was suggested that the virtual environment coupled with the cognitive task demands may have created an increase in cognitive load (i.e., more demand). Thus, exercise per se was not the cause of upregulated P3 responses. Considering a lack of consensus on the exercise–cognition relationship, future research examining exercise dose and cognitive domain variables is warranted.

As with any study examining the influence of exercise on cognitive function, there are several potential limitations worth mentioning. First, subjects performed faster on frequent trials but less accurately on rare trials, which may have been due to boredom associated with the length of the testing sessions (50 min). Over time, participants may lose focus and start anticipating the presentation of a frequent stimulus. The improvements in reaction time during frequent trials are likely due to the reduction in accuracy during rare trials (i.e., speed–accuracy tradeoff). We did not include a direct measure of boredom, focus, attention, or concentration that could have helped us determine how subjects were feeling over the course of each session. Future investigations may consider adding additional measures or active breaks that will counter the potential influence of boredom.

Second, the exercise duration and intensity may not have been long or difficult enough to have a positive effect on the primary outcome measures. For example, a meta-analysis by Lambourne and Tomporowski (2010) suggests that impairments in cognitive performance occur during the first 20 min of exercise regardless of intensity. However, following impairments observed from 0 to 20 min, general improvements in cognition are found. Therefore, a 20 min bout of low-intensity exercise may have been too short to provide a beneficial effect on cognitive function. Researchers should consider examining dose-response relationships between exercise duration, intensity, and cognitive function. Additionally, incorporating other potential moderators that influence the cognition–exercise relationship (e.g., exercise type, exercise frequency) is important for future research.

Third, the addition of EEG artifact or skin potentials could have affected P3 amplitude and latency responses. Over time, especially during exercise, skin potentials are likely to occur due to perspiration and heat. This not only creates the possibility of skin potentials but may also lead to bridging between electrodes. To reduce the likelihood of this occurring, the recording chamber uses an isolated air conditioning unit and thermostat that was used to keep the room at a stable temperature and humidity throughout exercise. Additionally, the electrode gel that is used for recording is highly viscous and remains solid under exercise conditions. Finally, very careful attention was taken during the data collection and processing steps. Participants sat on a recumbent bike during both recording sessions. This seated posture provides back support and allows clearance for the EEG electrode wire harness. This position also reduces the sway of the neck, torso, and shoulders. During data processing, a semi-automated procedure was implemented whereby researchers visually inspected continuous and segmented data to ensure movement artifact was kept to a minimum. All remaining data quality standards (e.g., artifact detection settings, blinding of researchers to conditions) were maintained and implemented throughout the data processing procedures.

## 5. Conclusions

Together, results from the current investigation suggest low-intensity exercise exerts small-to-negligible effects on behavioral performance measures of accuracy and reaction time but may lead to functional differences, indexed by P3, may occur during exercise. However, these functional differences may not be sufficient enough to alter behavioral outcomes during this type of cognitive task. This study adds to the small but growing body of literature that examines changes in cognitive performance during steady-state exercise. The results that were found are contrary to many similar studies in the area. With the observed similarities in response accuracy and reaction time between conditions, low-intensity exercise may not have as large of an effect on cognition as previously thought. The reductions in P3 amplitude during exercise also oppose much of the existing literature, though few of the studies exclusively examine low-intensity exercise.

## Figures and Tables

**Table 1:** Participant Characteristics (M ± SD) Overall and by Sex.

Measure	Male (*n* = 17)	Female (*n* = 10)	Total (N = 27)
Age (years)	23.6 ± 3.4	21.6 ± 1.3	22.9 ± 3.0
BMI (kg/m^2^)	25.5 ± 3.3	25.2 ± 4.7	25.4 ± 3.8
Depressive Symptoms (BDI) ^1^	3.9 ± 3.5	6.0 ± 5.1	4.7 ± 4.2
Anxiety levels (STAI)	47.3 ± 3.7	46.6 ± 2.3	47.0 ± 3.2
Perceived Stress (PSS)	27.7 ± 5.7	31.2 ± 4.0	29.0 ± 5.3

^1^ Significant difference, unpaired Student’s *t* test between male and female participants, *p* < 0.05.

## Data Availability

The data presented in this study are available on request from the corresponding author. The data are not publicly available due to privacy and ethical reasons.
